# Stress, anxiety, and social support among pregnant Japanese women: A qualitative study

**DOI:** 10.18332/ejm/226323

**Published:** 2026-10-26

**Authors:** Yoshiko Abe, Noboru Iwata, Midori Kikuchi, Gen Kobashi

**Affiliations:** 1 Department of Public Health, Dokkyo Medical University, Tochigi, Japan; 2 Graduate School of Nursing, Dokkyo Medical University, Tochigi, Japan; 3 Health Center, Dokkyo Medical University, Tochigi, Japan

**Keywords:** pregnancy, anxiety, stress, social support, qualitative study

## Abstract

**INTRODUCTION:**

No study has evaluated social support in the context of stress and anxiety management during pregnancy. This study aimed to explore stress and anxiety types experienced by Japanese women during pregnancy and the social support types for coping with them.

**METHODS:**

An exploratory qualitative study was conducted to gain details of the stress, anxiety, and social support experienced during pregnancy. Data collection was conducted between January and April 2024, and involved a total of four focus group interviews with 10 participants recruited via convenience sampling. Thematic analysis was performed using the qualitative software package NVivo 11.

**RESULTS:**

The participants’ mean age was 32 years; three were multiparous, seven were primiparous. This study identified that ‘physical’ and ‘life changes’ due to pregnancy cause various stresses and anxieties. Emotional and appraisal support combine to emotionally reduce stress and anxiety throughout pregnancy, whereas instrumental and informational support seem to function by removing the causes of stress and anxiety. These four types of support were offered as ‘support sought’ and ‘support received’, and it was found that each could also serve as a source of different stress and anxiety.

**CONCLUSIONS:**

This study indicates that Japanese pregnancy-related stress and anxiety stem from ‘physical changes’ and ‘lifestyle changes’, and that pregnant women cope with these multifaceted stressors by utilizing four types of social support. Future research examining details specific to each stage of pregnancy and incorporating the perspectives of support providers would yield valuable insights for establishing more effective support systems.

## INTRODUCTION

Stress and anxiety are common conditions experienced during pregnancy and are associated with an increased risk of mental disorders, such as perinatal depression^1^, which are associated with various adverse perinatal outcomes, such as preterm birth and low birth weight, as well as offspring development^2,3^. Pregnancy introduces severe uncertainties and fears regarding childbirth, fetal health, and bodily changes, which significantly elevate maternal stress and anxiety^4^. Strong social support, such as psychological care, medical support, and reliable information, are acts as a vital buffer^5^; conversely, a lack of social support significantly increases the risks of antenatal depression, anxiety, and preterm birth, while also hindering recovery from perinatal depression^6,7^.

According to the transactional theory of stress and coping, when facing stressors in daily life, constantly appraise whether they are manageable, then if the stimuli are perceived as harmful and exceed an individual’s capacity to cope, social support needs to be mobilized^8,9^. Moreover, Cronenwett^10^ stated that the transition to parenthood impacts structural and functional support owing to altered needs for social support and restructured social networks. In addition, pregnant women differ individually in the quality and quantity of social support they require for this life transition^10^. As defined in the American Psychological Association dictionary, stress is ‘the physiological or psychological response to internal or external stressors’^11^, and anxiety is ‘an emotion characterized by apprehension and somatic symptoms of tension in which an individual anticipates impending danger, catastrophe, or misfortune’^12^. Therefore, they are considered to trigger different types of coping mechanisms and require different social supports. However, most of previous studies have conflated the stress and anxiety experienced during pregnancy and have failed to evaluate the detailed role of social support^13^. Therefore, understanding of pregnancy-related stress and anxiety, and clarifying the specifics of the social support that addresses them, can lead to the provision of more appropriate care. Furthermore, given that social support is shaped by social and cultural environments, understanding the experiences and perceptions of pregnant women in the Japanese context – including the various stressors and anxieties they face and the types of social support they require – has the potential to foster better pregnancy and childbirth experiences.

Therefore, this study aimed to explore the types of stress and anxiety experienced by pregnant Japanese women and the social support types for coping with them.

## METHODS

### Design

An exploratory qualitative design was used. Ethical approval for this study was obtained from the Bioethics Committee of Dokkyo Medical University (2023-008). This study was conducted in accordance with the Declaration of Helsinki and relevant guidelines and regulations. Written informed consent was obtained from all the participants before the interviews.

### Participants

Participants were recruited using convenience sampling from among mothers who underwent a 10-month infant checkup in a town, Tochigi, Japan. To obtain information about their experiences and feelings toward social support throughout pregnancy, we approached mothers after delivery. Japanese mothers older than 18 years were included, and those with mental health problems were excluded. The mothers, the interviewer, and another researcher were present at the interview.

### Procedures

After recruitment, the participants received an explanation of the study, including confidentiality and autonomy, in a private room. All participants provided written informed consent. Semi-structured interviews were conducted using a researcher-developed topic guide with open-ended questions about thoughts and feelings regarding experiences of stress and anxiety during pregnancy and social support types. Stress was assessed by asking, ‘What kind of mental and physical stress did you experience during your pregnancy?’. Anxiety was determined using the question ‘What kind of anxieties did you experience during your pregnancy?’. At the beginning of the interview, following explanations were also added: for stress, ‘changes in physical condition and the accompanying experience of irritability’, and for anxiety, ‘worries such as whether the baby is okay’. On their experience of social support, they were asked, ‘What do you think has helped you deal with stress/anxieties?’. A female researcher (YA), a public health researcher with a background in pediatrics and skilled in conducting focus group interviews with mothers, facilitated the interviews. A female psychologist (MK) attended the interviews to observe the participants’ nonverbal behaviors. During the interviews, all participants were assigned a code to maintain confidentiality. The interviews lasted approximately 40–60 min, and all interviews were recorded. The researcher created field notes documenting aspects such as the participants’ non-verbal communication (e.g. facial expressions and gestures). Data collection was conducted during the monthly 10-month infant checkup between January and April 2024. During the data collection, thematic saturation was monitored through concurrently conduct data collection and thematic analysis using an iterative approach. Since the fourth focus group (total sample size of 10) yielded no novel codes or overarching themes, it was determined that data saturation was achieved.

### Data analysis

A thematic analysis was performed^14^. The recorded interviews were transcribed, stress and anxiety were inductively analyzed^15^, and social support was deductively analyzed using the existing concepts of social support. Social support was conceptualized as either structural or functional. Structural social support refers to ‘the existence and quantity of social relationships (e.g. marital status, group membership, and the number of friends one has) and the interconnectedness of a person’s social relationships or social network (e.g. the degree to which a person’s friends know each other)’. Moreover, functional social support is the degree to which interpersonal relationships serve particular functions^16^. Functional support was conceptualized into four defining attributes: emotional, instrumental, informational, and appraisal^17^. Since priority was placed on the participants’ raw, initial spoken accounts, the interview transcripts were not returned to the participants. Instead, the researcher verified the content and obtained confirmation at the end of the interview. YA repeatedly read the transcripts until the author became familiar with the content. Subsequently, direct words or phrases with similar meanings from the transcripts were identified and condensed into codes. These codes were further condensed and organized into categories, which were finally grouped into overarching themes. This data analysis was conducted iteratively throughout the data collection period, involving discussions among researchers, until no new categories emerged^18^. YA had no prior relationship with any of the participants before the study. The participants were informed that the interviewer was a public health researcher, and about the specific research aims for the first time at the start of the interview. The other authors (MK and NI) reviewed the coding and discussed the final themes. Any discrepancies in coding or theme generation were resolved through discussion and consensus among the research team until a final agreement was reached. The qualitative software package NVivo 11 (QSR International Pty Ltd., Victoria) was used for transcription, coding, and management of themes. The reporting of this study followed the COREQ guidelines (Supplementary file).

## RESULTS

### Participant characteristics

The characteristics of the 10 participants are presented in Table 1. Their mean age was 32.1 years (range: 26–40); three were multiparous, seven were primiparous; none had multiple births. All lived with their husbands and child (children); nine continued to work during pregnancy; eight were regular employees, and one had a part-time job. One participant was a housewife before pregnancy.

**Table 1:** Participant characteristics of qualitative study about stress, anxiety, and social support among pregnant women, conducted at a 10-month infant checkup in Tochigi, Japan, January – April 2024 (N=10)

*ID*	*Age (years)*	*Primipara/Multipara*	*Number of fetuses*	*Current family members*	*Currently working*	*Current work status*	*Work during pregnancy*	*Work status during pregnancy*	*When maternity leave was commenced (weeks of gestation)*
P1	40	Multipara	1	Husband, child	Yes	Childcare leave	Yes	Regular employee	33
P2	38	Multipara	1	Husband, child	No	-	No	-	-
P3	30	Primipara	1	Husband, child	Yes	Childcare leave	Yes	Regular employee	34
P4	26	Primipara	1	Husband, child	Yes	Childcare leave	Yes	Regular employee	34
P5	27	Primipara	1	Husband, child	Yes	Childcare leave	Yes	Regular employee	34
P6	30	Primipara	1	Husband, child	Yes	Childcare leave	Yes	Regular employee	36
P7	34	Primipara	1	Husband, child	Yes	Childcare leave	Yes	Regular employee	32
P8	30	Primipara	1	Husband, child	Yes	Working	Yes	Regular employee	34
P9	37	Multipara	1	Husband, children	No		Yes	Part-time job	33
P10	29	Primipara	1	Husband, child	Yes	Childcare leave	Yes	Regular employee	32

The mean week of gestation when commencing maternity leave among working pregnant women was 30.2 (range: 32–36) weeks. Most participants were still on childcare leave, and only one had resumed work at the time of the interview.

### Perceived stress during pregnancy

We extracted four themes that explained the experience of stress during pregnancy: ‘stresses caused by physical changes’, ‘stresses caused by life changes’, ‘stresses related to seeking support’, and ‘stresses related to not getting support’. Table 2 lists the themes, categories and sample quotes. The ten participants are coded P1–P10, for anonymity.

**Table 2:** Themes, categories, and sample quotes on stress types experienced during pregnancy identified through thematic analysis of four focus groups of qualitative study about stress, anxiety, and social support among pregnant women, conducted at a 10-month infant checkup in Tochigi, Japan, January – April 2024 (N=10)

*Theme*	*Category*	*Quotes*
**Stresses caused by physical changes**	Morning sickness	*‘My morning sickness was pretty bad, so I couldn’t move my body the way I wanted to. That was the hardest part.’* (P3)
	Minor troubles	*‘I had a lot of minor problems, like a eustachian tube disorder, or something to do with my ears. Yes. I had some kind of minor problem, and it was pretty hard to go to work.’* (P3)
	Difficulty in movement	*‘Compared to my first pregnancy, maybe it’s because I’m getting older, but I didn’t realize my stomach had been so heavy during pregnancy. It was just hard to move around.’* (P2)
**Stresses caused by life changes**	Cannot do what she used to be able to do	*‘My morning sickness was pretty bad and I would throw up and stuff. So, I couldn’t cook or clean. My husband helped a little, but he didn’t clean much, so the house just got dirtier and dirtier. I felt frustrated because I couldn’t do much myself and it made me feel sick.’* (P5)*‘I was feeling bloated and sleepy, so I couldn’t finish what I had been hoping to do.’* (P6)
	Limit what she can do	*‘I usually relieve stress by eating delicious food or going out, but there was a time when I couldn’t do that and I just got deeper anddeeper into a rut.’* (P7)
**Stresses related to seeking support**	Caring about the family’s reactions	*‘I had morning sickness and needed to get an IV drip at a clinic, but only my mother and husband could drive, so I had to rely on one of them. It was pretty tough, so I thought it would be helpful if there was something like a taxi.’* (P3)Interviewer: ‘Asking help to family might be easy, but at the same time did you feel something uncomfortable?’*‘Yes. If they say they’re busy that day, I feel kind of sorry.’* (P3)
	Caring about colleagues’ reactions	*‘I was hospitalized for quite a long time, so yes. I found it difficult to take time off work or call my boss.’* (P3)
**Stresses related to not getting support**	Lack of consideration from husband	*‘Up until now, I’ve been doing most of the housework at home, so that was fine, but when I became pregnant, it took a toll on my body, so even if it wasn’t a full-on share, I wish there had been some kind of consideration, or that my husband would’ve done a little, but I didn’t really get that, so I felt quite stressed.’* (P4)
	Cannot talk to husband	*‘My husband was away on a business trip for a long time and came over on the weekends. I was a little sad about it, but he was the easiest person to tell, so it was stressful not being able to tell him how sad I was because telling him in person and telling him over LINE or phone is completely different.’* (P7)
	Lack of consideration from workplace	*‘Also, I continued working so my workplace was understanding, but people who didn’t have any children at all, you know, they would give me an attitude like, “Why are you having such a hard time? Can’t you work more?”. It was a bit stressful.’* (P1)
	Insensitive words by medical professionals	*‘I had a really hard time managing my weight, and my morning sickness turned into eating disorders. Every time I went to the hospital, the doctor would get angry at me for eating too much, and I remember that being really scary.’* (P9)

A thematic analysis using the qualitative software package NVivo 11 (QSR International Pty Ltd., Victoria) was performed.

### Stresses caused by physical changes

Most participants reported stress caused by poor health conditions, such as morning sickness, minor troubles, and difficulty moving:

*‘My morning sickness was pretty bad, so I couldn’t move my body the way I wanted to. That was the hardest part.’* (P3)

### Stresses caused by life changes

Additionally, the poor health conditions left her so stressed that she could no longer do what she was able to do before or limited what she could do:

*‘My morning sickness was pretty bad, and I would throw up and stuff. So, I couldn’t cook or clean. My husband helped a little, but he didn’t clean much, so the house got dirtier. I felt frustrated because I couldn’t do much myself, and it made me feel sick.’* (P5)

### Stresses related to seeking support

Although they seek support to manage the inconveniences experienced during their pregnancy, seeking support necessitates consideration of others from whom they require support, such as concerns for families’ and colleagues’ reactions to their condition:

*‘I had morning sickness and needed to get an IV drip at a clinic, but only my mother and husband could drive, so I had to rely on one of them. It was really tough, so I thought it would be helpful if there* was *something like a taxi.’* (P3)

Interviewer: ‘Asking help from family might be easy, but at the same time, did you find something uncomfortable?’:

*‘Yes. If they say they are busy that day, I feel sorry* (*for myself*).’ (P3)

### Stresses related to not getting support

However, when the support provided by others did not match their expectations, such as the lack of consideration by the husband, inability to talk to the husband, lack of consideration from colleagues, and insensitive words by medical professionals, it caused stress:

*‘I continued working during pregnancy, and my workplace was understanding, but people who didn’t have any children, you know they would give me an attitude like, “Why are you having such a hard time? Can’t you work more?”. It was a bit stressful.’* (P1)

### Perceived anxiety during pregnancy

Four themes emerged to explain perceived anxiety: anxiety caused by physical changes, anxiety caused by life changes, anxiety related to seeking support, and anxiety related to not receiving support. Table 3 lists the themes, categories and sample quotes.

**Table 3:** Themes, categories, and sample quotes on anxiety types experienced during pregnancy identified through thematic analysis of four focus groups of qualitative study about stress, anxiety, and social support among pregnant women, conducted at a 10-month infant checkup in Tochigi, Japan, between January and April 2024 (N=10)

*Theme*	*Category*	*Quotes*
**Anxieties caused by physical changes**	Health of infant	*‘Before I noticed any fetal movement, I was always worried that the baby was not alive because there was no movement, but there was nothing I could do by worrying, so I held off until my checkup and went to the doctor. I was relieved that the baby was moving, and just went about my daily life. After I started to notice the baby moving, I knew it was moving, but I was quite bloated and it was painful towards the end, so I didn’t know how much I should rest.’* (P4)
	Minor troubles	*‘I felt anxious because it didn’t like a threatened preterm birth that I needed to receive any medical treatment or anything like that, and I had to endure it and investigate it myself. I wasn’t in a state where I needed to receive an IV drip or anything, but I was worried about how many more months this would last.’* (P1)
**Anxieties caused by life changes**	Concerns about childbirth and postpartum	*‘Everyone was saying it [delivery] hurt so I was quite scared of the pain and I kept asking people what it felt like.’* (P8)*‘If you ask people who’ve had it, they’ll say that it’s going to be ok, but I was worried about how to do it actually.’* (P9)*‘I wondered what would happen to them after they were born, what everyone else was doing, and how I would give them both a bath, thinking that things would work out somehow once they were born, and thinking about little things like that.’* (P2)*‘What would I do if they both started crying at the same time?’* (P1)*‘If you ask people who’ve had experience, they say it will work out somehow, but I was worried about how I would do it.’* (P1)
	Fear of COVID-19	*‘The COVID-19 situation was much worse back then, so I was afraid to go to places like that as a pregnant woman. I have an older child, so I wanted to take him to a support center or something, but I was a little scared.’* (P2)
**Anxiety related to seeking support**	Information overload	*‘I search for information on the internet, but in the end, I’m left with other concerns, like, “Oh, this could happen, too’’.’* (P8)*‘How can I say* … *They were written in such a bad way that some of them were written in great detail, but some of them were written in a lighter light way, and I was worried after reading both of them.’* (P4)
**Anxiety related to not getting support**	Insufficient information	*‘People with experience told me it would be fine. But I was worried why or whether I would be able to do it.’* (P1)

A thematic analysis using the qualitative software package NVivo 11 (QSR International Pty Ltd., Victoria) was performed.

### Anxiety caused by physical changes

Physical changes caused by pregnancy include minor troubles and concerns that pregnant women have about the condition of their infants:

*‘I was not in preterm birth or anything that required medical attention, so I felt anxious that I had to endure and investigate it myself. I wasn’t in a state where I needed to receive an IV drip or anything, but I was worried about how many more months this would last.’* (P1)

### Anxiety caused by life changes

Physical changes bring about several life changes that cause anxiety regarding childcare during pregnancy, childbirth, and after delivery. Additionally, since their pregnancy occurred during the coronavirus disease 2019 (COVID-19) pandemic, fear of COVID-19 infection was also experienced by the participants:

‘*Everyone was saying it [delivery] hurt so I was quite scared of the pain and I kept asking people what it felt like.’* (P8)

*‘The COVID-19 situation was much worse back then, so I was afraid to go to places like that as a pregnant woman. I had an older child, so I wanted to take him to a support center or something, but I was a little afraid.’* (P2)

### Anxiety related to seeking support

Most participants sought information through the internet to alleviate their anxiety. However, this can also cause information overload and vice versa, which in turn can cause anxiety:

*‘I search for information on the internet, but in the end, I’m left with other concerns, like, “Oh, this could happen, too”.’* (P8)

### Anxiety related to not getting support

When the information received was insufficient, participants tended to feel anxious:

*‘People with experience told me it would be fine. However, I was worried about why and whether I would be able to do it.’* (P1)

Overall, stress and anxiety during pregnancy are caused by two types of changes: physical and lifestyle. However, seeking support to overcome this stress and anxiety led to mental fatigue, frustration, and information overload, which caused further stress and anxiety (Figures 1 and 2).

**Figure 1:**
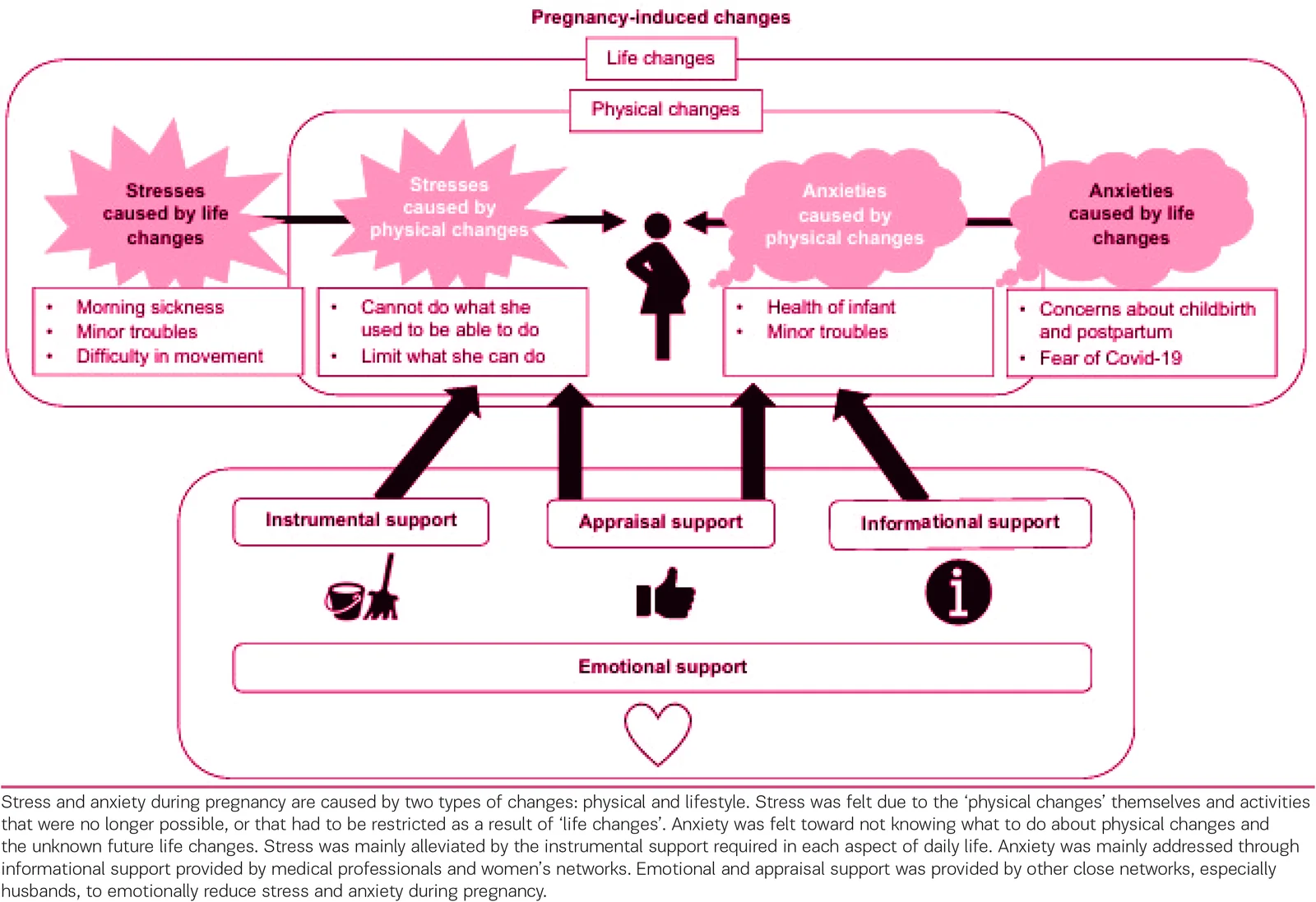
Stress, anxiety, and experienced social support during pregnancy identified through a qualitative study about stress, anxiety, and social support among pregnant women, conducted at a 10-month infant checkup in Tochigi, Japan, January–April 2024 (N=10)

**Figure 2:**
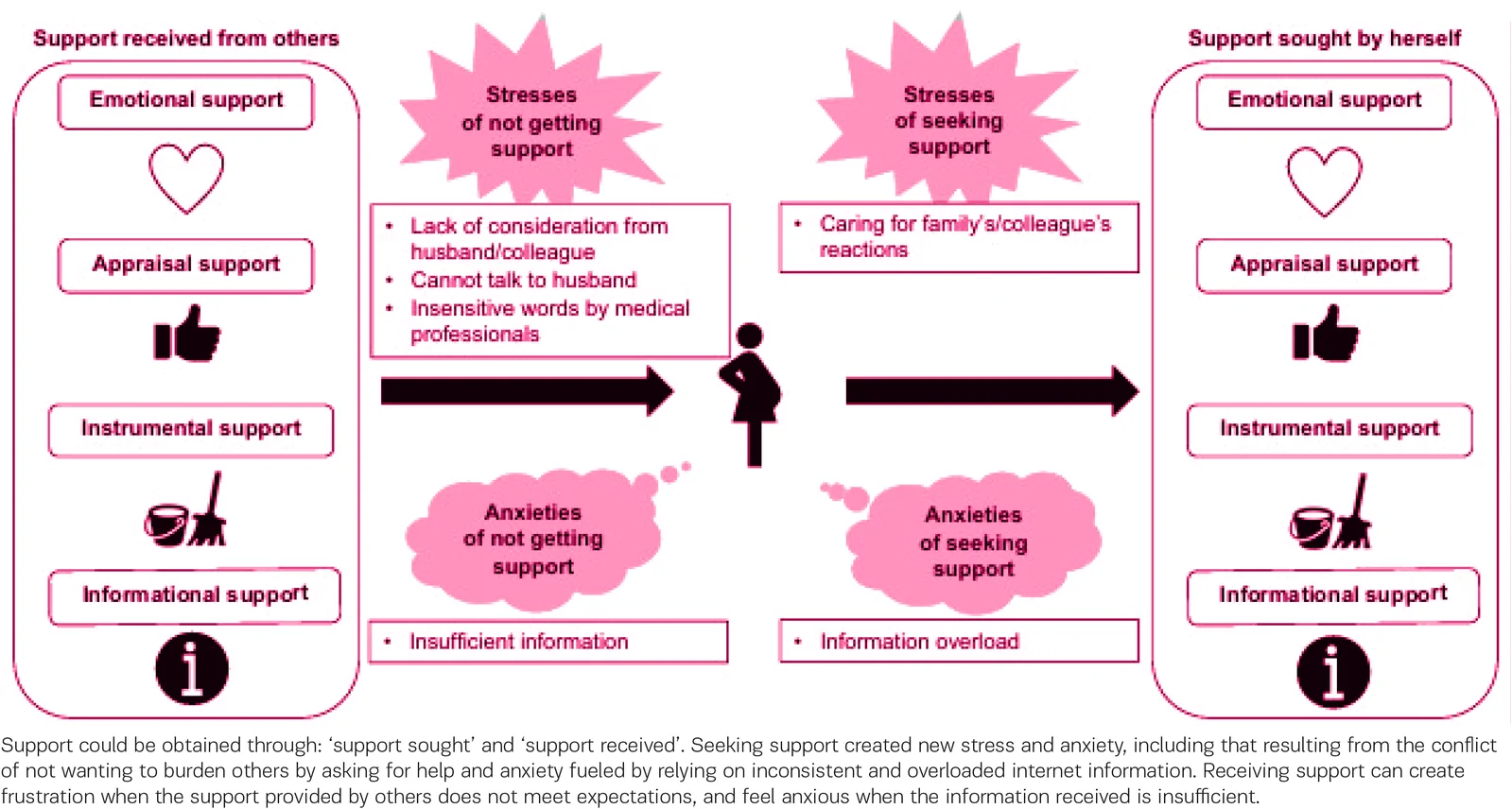
Stress and anxiety caused by seeking and receiving support identified through a qualitative study about stress, anxiety, and social support among pregnant women, conducted at a 10-month infant checkup in Tochigi, Japan, January–April 2024 (N=10)

### Perceived social support

The themes, categories, and sample quotes on social support types experienced during pregnancy are presented in Table 4.

**Table 4:** Themes, categories, and sample quotes on social support types experienced during pregnancy identified through thematic analysis of four focus groups of qualitative study about stress, anxiety, and social support among pregnant women, conducted at a 10-month infant checkup in Tochigi, Japan, between January and April 2024 (N=10)

*Theme*	*Category*	*Quotes*
**Emotional support**		*‘I’m quite a negative person, but my husband is quite positive. He cheers me up by telling me that it’s okay even when I get worried, and it helps me a lot. He also asks me every day what I can eat.’* (P3)*‘When I was feeling anxious, he listened to me. When I said something like, “I’m having a lot of work to do, what should I do?”, he would say, “Maybe you should just turn it off a little”. When I was feeling negative, he would say, “Why don’t you go for a walk?” and bring me to a more positive side. Those were the things that helped me the most.’* (P6)*‘There were a lot of fathers with children at work. They would ask me all sorts of questions about the difficulties their wives were going through during pregnancy, like, “Are you OK?” and “Are you pushing yourself too hard?” and would ask me all sorts of questions several times a week. I felt like it was really easy to tell them when I was feeling down.’* (P6)
**Appraisal support**		*‘Isn’t pregnancy a pretty delicate thing? There were people who were trying to get pregnant, but when I got pregnant, I faced criticism and couldn’t stand it* … *[my doctor] told me that I should take a month off work* … *He said that I could start work again after the New Year’s holiday and with a fresh mind in the new year, and that was really reassuring, and I felt like it was okay to take a break.’* (P7)
**Instrumental support**	Housework support	*‘I got home early, so I cooked the weekday meals, and my husband did all the shopping and dinner on weekends, so it was really easy. Even when I was feeling nauseous and wanted to stay in bed all day, he would prepare dinner by himself and call me when it was ready. That was really helpful.’* (P8)*‘I was living at my parents’ house at the time, so it was really helpful that I didn’t have to worry about cooking or do any housework.’* (P3)
	Work support	*‘Because it’s a standing job, the people around me were very considerate and I was allowed to do clerical work as much as possible, so I didn’t feel that stressed about work.’* (P5)
	Childbirth and nursing preparation	*‘*… *she said she’d lend it to me after she’d finished everything, but when she lent it to me, I was like, thank you, thank you. But once things had settled down, I went to (a shop of baby materials) and was shocked at the prices.* *She lent me things like I’d never lent her before, and she really helped me financially.’* (P7)
	Childbirth and nursing preparation classes	*‘I wish my husband had participated [in a child preparation class]. He might be able to do it, but I think it would have been nice if there had been some kind of opportunity like that …’* (P3)*‘I asked a lot of questions before the birth, not just from the local government but also from the hospital, but I think it would have been easier to understand if the whole class, not just to the mother, but to both parents were basically in attendance. I also watched a video, and the scene when the baby was born was quite a shock, so I think if the husband watched it with the mother, it would help him prepare himself a little better.’* (P6)
	Transportation	*‘I was sick and couldn’t drive, so I thought it would be nice if there was a demand taxi service that I could use to take me to and from the hospital without worrying. I had morning sickness and only my mother and husband could drive, so I had to rely on either of them. It was pretty tough, so I thought it would be helpful to have a taxi service.’* (P3)
**Informational support**	Pregnancy experience	*‘There are people around me at work who have experience raising children, and they tell me things like, for example, it was like this when they were raising children, and this was tough, but now my kids are so grown up, so it’ll be okay. That kind of encouragement from people with experience can really calm me down.’* (P1)
	Medical information	*‘The obstetrics and gynecology clinic I went to had a doctor who was very considerate, so I felt like I could ask the doctor if I had any concerns.’* (P5)
	Childbirth preparation information	*‘When I go to [a shop for baby items], there are catalogs for preparing for childbirth and childcare, and I think, “Oh, I need this, too”, but they tell me that I don’t need about half of them. If I don’t know, I’ll end up asking myself, “How much would it cost to buy everything?”. But they tell me what I don’t need.’* (P7)

A thematic analysis using the qualitative software package NVivo 11 (QSR International Pty Ltd., Victoria) was performed.

### Structural support

The structural support network of our participants is shown in Figure 3. Based on the focus group interviews, the network members of the participants comprised family members (mainly husbands, mothers, and mothers-in-law), friends and colleagues, connections through internet/social networking sites (SNS), medical staff, and local government staff. They spoke most frequently with their husbands and had close relationships with them; as well as with their mothers, with whom they had similar relationships. They also met and spoke frequently with friends and colleagues; however, their intimacy with these individuals was weaker than that with their family. They used the internet and SNS almost every day, but their relationships were one-sided. Opportunities to meet and talk with medical staff and fellow members of the local government were limited, and intimacy was also limited.

**Figure 3:**
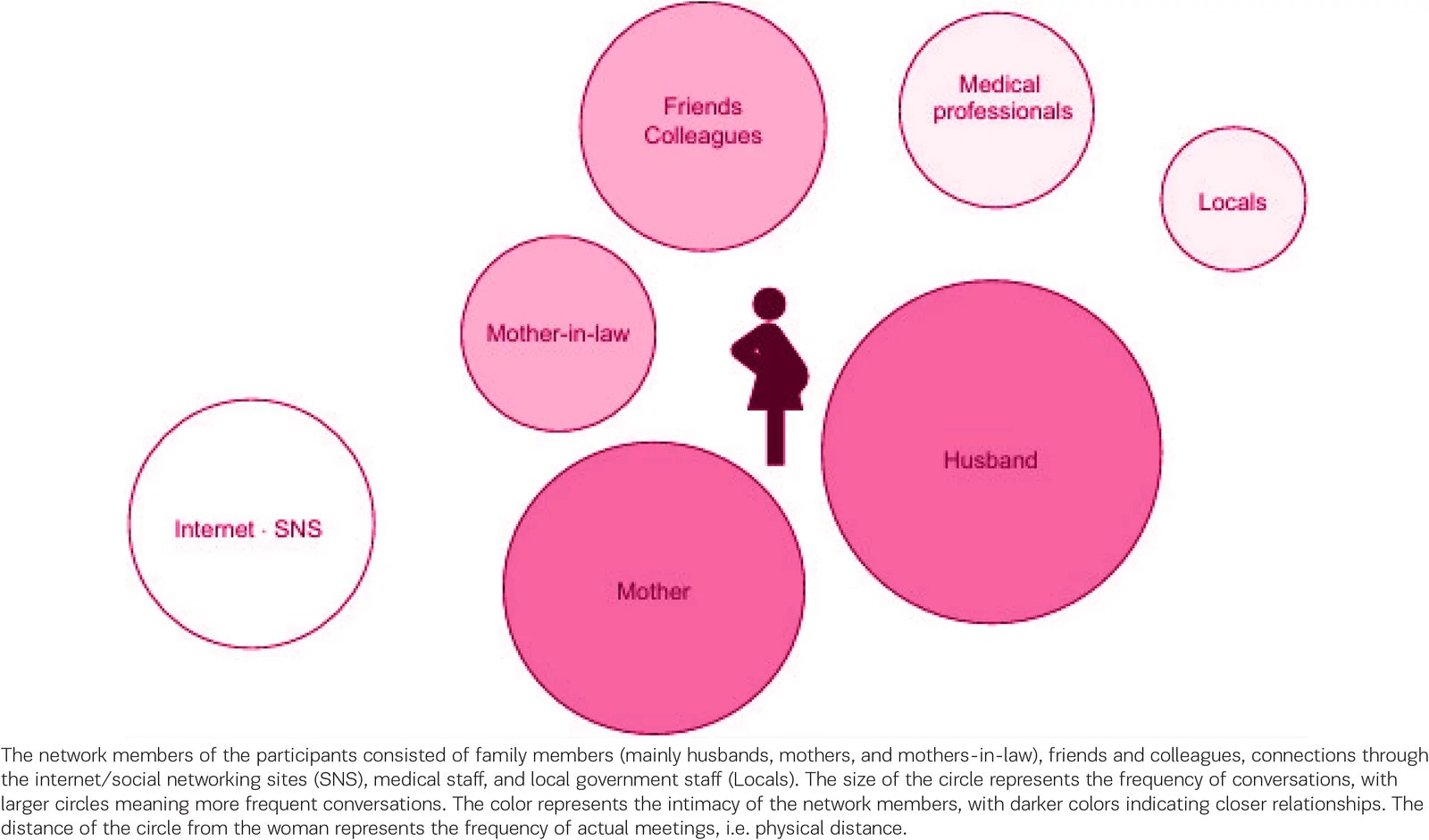
Structural social support identified through a qualitative study about stress, anxiety, and social support among pregnant women, conducted at a 10-month infant checkup in Tochigi, Japan, January–April 2024 (N=10)

### Emotional support

This support was mainly provided by the husbands. Women’s networks, including their mothers, friends, and colleagues, were also the main sources of emotional support:

*‘I’m quite a negative person, but my husband is quite positive. He cheers me up by telling me that it’s acceptable even when I get worried, and this helps me a lot.’* (P3)

*‘We all had pregnancy around the same period, so if I ask them, they’ll tell me anything. I think I consulted with my friends and colleagues the most.’* (P8)

### Appraisal support

Feeling that others understood their difficult times during pregnancy and acknowledged their perseverance was a strong source of support for participants:

*‘Isn’t pregnancy a pretty delicate thing? There were people who were trying to get pregnant, but when I got pregnant, I faced criticism and couldn’t stand it* … *[my doctor] told me that I should take a month off work* … *He said that I could start work again after the New Year’s holiday and with a fresh mind in the new year, and that was really reassuring, and I felt like it was okay to take a break.’* (P7)

### Instrumental support

This support type was divided into five categories based on life situations: housework support, work support, childbirth and nursing preparation, childbirth and nursing preparation classes, and transportation. Housework support from the husband and mother was necessary to manage stress when she could not do what she was used to doing. Similarly, work support from colleagues was required to ease the stress of what they had not been able to do. In terms of childbirth preparation, borrowing or obtaining necessary items from female friends supported not only preparation but also alleviated financial burdens. Many participants required childbirth and nursing preparation classes to develop their husbands’ understanding of pregnancy and childbirth. Additionally, transportation was required to resolve transportation difficulties due to pregnancy:

*‘I got home early, so I cooked the weekday meals, and my husband did all the shopping and dinner on weekends, so it was really easy. Even when I felt nauseous and wanted to stay in bed all day, he prepared dinner himself and called me when it was ready. This was really helpful.’* (P8)

*‘Because it’s a standing job, the people around me were very considerate and I was allowed to do clerical work as much, so I didn’t feel that stressed about work.’* (P5)

*‘[A friend who had given birth] would lend me some childcare items that they no longer needed … It helped me out a lot financially, too.’* (P7)

### Informational support

Support was divided into three categories based on the type of information: pregnancy experience, childbirth preparation, and medical information. A female network, including mothers and friends, is one of the most reliable and easy-to-obtain information sources. However, in terms of information about infants’ conditions, they sought evidence-based information to alleviate anxiety. Information on childbirth preparation reduces the difficulty of selecting the many items needed. Additionally, most participants used the internet and SNS to obtain this type of information because they could easily obtain their desired answers through these platforms:

*‘When someone who has experience says: “It’s okay”, and someone who doesn’t have experience says it based on just some mobile information, it’s a big deal, and it’s completely different* … *I can understand without doubt (if mother or mother-in-law said it).’* (P7)

*‘I think it would be nice if there was a program where on could ask for advice from people with medical knowledge, like midwives or public health nurses, in close contact.’* (P1)

Overall, emotional support and appraisal support were mainly provided by the husband or close female network members, and it was essential source of support to emotionally mitigate several stress and anxiety. Instrumental support seems to well work to reduce stress caused by physical change and life change, such as support for housework and work. Informational support might be effective to ease anxiety caused by unknown future, such as listening to other experiences, and obtaining medical information (Figure 1, Table 4).

## DISCUSSION

This study illustrated that the ‘physical’ and ‘life changes’ caused by pregnancy led to stress and anxiety in Japanese pregnant women in various ways. Stress was felt due to the ‘physical changes’ themselves and activities that were no longer possible, or that had to be restricted as a result of ‘life changes’, which was mainly alleviated by the instrumental support required in each aspect of daily life. Anxiety was felt toward not knowing what to do about physical changes and the unknown future life changes, which was mainly addressed through informational support provided by medical professionals and women’s networks. Additionally, emotional and appraisal support was provided by other close networks, especially husbands, to reduce stress and anxiety during pregnancy. Support could be obtained through: ‘support sought’ and ‘support received’. Seeking support created new stress and anxiety, and receiving support can create frustration when the support provided by others does not meet expectations.

As defined in the American Psychological Association dictionary, stress is ‘the physiological or psychological response to internal or external stressors’^11^, and anxiety is ‘an emotion characterized by apprehension and somatic symptoms of tension in which an individual anticipates impending danger, catastrophe, or misfortune’^12^. Our participants might have recognized realities during pregnancy, such as morning sickness and minor problems or things they could no longer do after becoming pregnant as threats, leading to increased stress. As possible dangers or negative outcomes during pregnancy are often related to fetal problems and an unknown future after birth, participants might have responded to these with anxiety.

Currently, approximately 50% of pregnant women in Japan continue to work during pregnancy, and physical discomfort, fatigue, and the inability to perform household chores have been reported as major concerns associated with pregnancy^19^. Japan has a major pregnancy culture called ‘Satogaeri birth’, which involves returning to the parental home during pregnancy and receiving support from the parents. However, if they are unable to return due to their work, they must manage the burden of housework with their husbands. Many participants in this study also reported balancing housework and employment, and the lifestyle changes resulting from this social context might appear to be major sources of stress and anxiety. Additionally, work conditions, home environment, internal psychological factors, and hormones during pregnancy enhance uncontrollable stress, and increases feelings of low self-worth and self-esteem^20^. Moreover, the gap between ‘perfect or ideal pregnancy’ and ‘real pregnancy’ increases women’s sense of inadequacy and inability. Therefore, it is inferred that the stress and anxiety observed in this study stem from these psychological conflicts experienced by pregnant women due to the ‘physical changes’ and ‘lifestyle changes’.

Limited knowledge about their pregnant bodies and the labor process often leads to overconcern. To deal with these stresses and anxieties, our participants tended to prefer to judge whether their experiences are normal by sharing them with other female networks. Feeling understood and unjudged acceptance through appraisal support from close female networks and medical staff are crucial for women’s confidence^21,22^. Additionally, supports from their partners were the support they mostly told. The lack of emotional and instrumental partner support causes women to claim to have unwanted pregnancies^23^, increases the risk of perinatal depression^24^, and hinders women with depressive symptoms from seeking help^25^. Among our participants, instrumental and informational support seemed to function as a means to remove the causes of stress and anxiety. Instrumental support, including assistance with household chores, work, physical mobility, and prenatal care, is considered an essential component for the physical and emotional well-being of pregnant women, and its absence increases the risk of perinatal depression^26,27^. In Japanese society, women spend approximately four times as much time on housework and childcare as men^28^, and many working pregnant women are forced to balance work and housework responsibilities. In addition, although informational support and consultancy services exist in local governments, of the 30–40% of users, only 20–30% are satisfied with the services^19^. Although its absence is directly related to complaints and irritability, satisfactory support tends to involve gratitude and happiness^29^. Therefore, following current social changes, effective instrumental support from people around them is essential to manage mental conditions, and satisfactory instrumental support also leads to emotional support. In addition, informational support about pregnancy and delivery is highly valued^26^. In particular, medical staff is a preferred source of reliable information, and women mobilize as much information as possible using their female networks, providing reassurance, empathy, comfort, and acceptance^13^.

In our study, social support was obtained as ‘support sought’ and ‘support received’. Regarding ‘support sought,’ many participants reported feeling like a burden to their families and colleagues when seeking support, which was one of the stressors during pregnancy. Support seekers with internalized stigma tend to avoid asking for help directly, instead seeking help indirectly or avoiding it altogether to prevent guilt and shame, leading to lower levels of self-efficacy and self-esteem, and finally to loneliness and isolation^30,31^. To manage anxiety, readily available information is sought, such as SNS, which provides timely answers to participants’ concerns. However, information seeking may involve conflicting and inconsistent aspects, resulting in additional anxiety, stress, and confusion. In terms of ‘support received’, receiving unwanted support that does not meet participants’ expectations can be frustrating, tending towards great disappointment. Women’s internalized pregnancy-related stigma tends to make them perceive providers’ support as ineffective, affecting their communication.

By attempting to distinguish between stress and anxiety during pregnancy, this study described the details of the supports – specifically, who provides it and what kind of support is offered for particular types of stress or anxiety. These findings added new perspectives on the previous findings about stress, anxiety, and social support during pregnancy and may contribute to the development of effective support systems for them.

### Limitations

This study has some limitations. First, the small number per group may affect the accuracy and truthfulness of the findings and the extent to which the findings represent the focus of the study. Second, due to the characteristics of the participants, such as residential areas (suburban), generalization of the findings might be limited. Further research utilizing a more diverse sample across different regions or organizational contexts in Japan is needed to enhance the transferability of the findings. Third, since most of our participants were primiparas, our findings might be biased by primiparas perceptions. Fourth, we did not elicit themes related to the pregnancy stages from this study narrative. Therefore, the results do not capture changes throughout the course of pregnancy. Fifth, a convenience sampling method was used to recruit participants, which may have introduced selection bias. Sixth, because the interviews were conducted after delivery, the findings may be subject to recall bias. Seventh, the sensitive nature of the topic or the focus group interviews may have induced social desirability bias. Some participants might have hesitated to openly share feelings in front of other mothers.

## CONCLUSIONS

This study suggests unique stress and anxiety due to the ‘physical’ and ‘lifestyle changes’ that occur during pregnancy. They deal with these multifaceted stresses and anxieties using four different types of social support. The provision of support does not always solve stress and anxiety problems; therefore, it may be beneficial for support providers to understand the stress and anxiety behind the required support.

## Supplementary Material



## Data Availability

The data supporting this research are available from the corresponding author on reasonable request.
